# Cross-cultural adaptation and validation of the Italian version of the Hip disability and Osteoarthritis Outcome Score (HOOS)

**DOI:** 10.1186/s12955-018-0935-6

**Published:** 2018-06-04

**Authors:** Marina Torre, Ilaria Luzi, Fiorino Mirabella, Martina Del Manso, Gustavo Zanoli, Gabriele Tucci, Emilio Romanini

**Affiliations:** 1 National Centre for Clinical Excellence, Safety and Quality of Care, Istituto Superiore di Sanità, Viale Regina Elena 299, Rome, Italy; 2 Center for Behavioral Sciences and Mental Health, Istituto Superiore di Sanità, Viale Regina Elena 299, Rome, Italy; 3 Department of Infectious Diseases, Istituto Superiore di Sanità, Viale Regina Elena 299, Rome, Italy; 4 Casa di Cura Santa Maria Maddalena, Via Gorizia 2, Occhiobello, RO Italy; 5 Department of Orthopaedics and Traumatology, Ospedale S. Giuseppe, Via Olivella Km 1, Albano Laziale, RM Italy; 6 ArtroGruppo, Casa di Cura San Feliciano, Via Enrico De Ossò 6, Rome, Italy

**Keywords:** HOOS, Hip osteoarthritis, Total hip arthroplasty, Cross-cultural validation, Registry

## Abstract

**Objective:**

To create a translated version of the HOOS to fit the Italian population and to test its psychometric properties and validity in hip osteoarthritis (OA) patients undergoing total hip arthroplasty (THA).

**Design:**

The HOOS Italian version was developed according to published international guidelines that include preparation, forward translation and reconciliation, backward translation, review and harmonization, and proof reading. The Italian HOOS was administered to 145 patients (mean age 65.7 ± 11.6 years, 34–89, 58.6% women) undergoing THA. The following psychometric properties were evaluated: *internal consistency* (Cronbach’s alpha); *test-retest reliability* (Pearson’s *r* and intra-class correlation coefficient, ICC); *convergent validity* (Spearman’s rho between HOOS and SF-36); *responsiveness* (comparison of pre/post-THA scores, Wilcoxon signed rank test). Interpretability (floor and ceiling effects, skewness and kurtosis indexes) and acceptability (time to compiling, missing answers, and autonomy in compilation) were also evaluated.

**Results:**

Translation and transcultural adaptation were conducted in accordance with the international recommendation. The translation was deemed understandable and appropriate as to the transcultural adaptation. None of the patients reported to have met any difficulties in reading and understanding the HOOS items. Internal consistency and test-retest reliability were good for each HOOS subscale (Cronbach’s alpha ≥0.7, Pearson’s r and ICC > 0.80). Convergent validity showed the highest correlations (Spearman’s rho > 0.5) between HOOS and SF-36 subscales relating to similar dimensions. As to responsiveness, all HOOS subscales scores improved significantly after THA (*p* < 0.01). Interpretability was acceptable despite ceiling effect in post-THA assessment. Acceptability was good: HOOS resulted easy and quick to fill out (12 min on average).

**Conclusions:**

The HOOS was successfully cross-culturally adapted into Italian. The Italian HOOS showed good psychometric properties therefore it can be useful to assess outcomes in OA patients after THA. This study provided a basis for its use within the Italian Arthroplasty Registry and for future clinical trials.

**Electronic supplementary material:**

The online version of this article (10.1186/s12955-018-0935-6) contains supplementary material, which is available to authorized users.

## Background

Hip osteoarthritis (OA) is one of the major causes of chronic disability and has a significant impact on patients’ health-related quality of life (HRQoL). HRQoL assessment tools are based on patients’ opinion [patient-reported outcomes measures (PROMs)] and focus on the evaluation of physical, psychological and social functions, and well-being. The Hip disability and Osteoarthritis Outcome Score (HOOS) was developed in 2003 [1–3] to ask for the patients’ view about their hip limited functions. The HOOS is a validated self-administered questionnaire developed in English as an extension of the Western Ontario and McMaster Universities Osteoarthritis Index (WOMAC) [1, 2]_,_ therefore a comparison between the two is feasible. The HOOS, that was translated and validated in several languages [4–10], showed to be effective in measuring patient-relevant outcomes in OA patients even after THA, and more responsive than the WOMAC in younger and physically active patients [1, 2].

The main aims of the study were developing the Italian translation of the HOOS and evaluating its psychometric properties (internal consistency, test-retest reliability, convergent validity, and responsiveness), along with its interpretability and acceptability. In addition, based on other arthroplasty registries experience [11], the present analysis was also performed in the perspective of introducing HOOS in the setting of the Italian Arthroplasty Registry project (RIAP, www.iss.it/riap) [12].

## Method

The study was started as a line of research of the RIAP, which is funded by the Ministry of Health and coordinated by the Italian National Institute of Health [Istituto Superiore di Sanità (ISS)]. It was approved by the ISS Ethical Committee and organized in two steps. Firstly, the English HOOS was translated into Italian and adapted to the Italian setting. Secondly, its psychometric properties were assessed in a prospective study by testing internal consistency, test-retest reliability, convergent validity, and responsiveness. Interpretability and acceptability were also evaluated. COSMIN guidelines and checklist were used to verify the whole translation and validation process [13–15]. The data collected during the study were treated in compliance with the Italian legislation on personal data protection and sent to ISS in an anonymous form.

### Development of the Italian version of the HOOS: translation and cross-cultural adaptation

The translation process was performed according to the published international recommendations [16, 17] and arranged in six phases: preparation, forward translation, reconciliation, backward translation, review and harmonization, proof reading.

#### Preparation

The RIAP project leader and principal investigator of the study (MT) asked the HOOS developer Professor Ewa Roos to check if any other Italian translation was ongoing. Then, she received permission to use and translate the HOOS into Italian.

#### Forward translation and reconciliation

Two Italian mother tongue translators with a different professional background, a bioengineer (MT) and an orthopaedic surgeon (ER), independently translated the questionnaire into Italian. Then, a consensus meeting was organized so that the two translators could meet and agree upon a single shared version.

#### Backward translation

An English mother tongue translator having no specific background in the health field, and blinded to the original HOOS English version, back-translated the questionnaire into English.

#### Review and harmonization

A multidisciplinary committee that included the two translators (MT and ER) and another orthopaedic surgeon (GZ) with a documented expertise in questionnaire validation, reviewed and made the back-translation consistently. To discuss the final version, the existing Italian version of WOMAC [18] was also taken into account, since the original HOOS was partly derived from it. Finally, the committee agreed on a questionnaire that was checked against understandability and transcultural adaptation.

#### Proof reading

The last agreed questionnaire was administered to a subgroup of seven OA patients undergoing THA for cognitive debriefing, to test alternative wording and check understandability, interpretation, and cultural relevance of the translation. After the last review, the final Italian HOOS was released.

### Patient enrollment and questionnaires administration

**Fig. 1:**
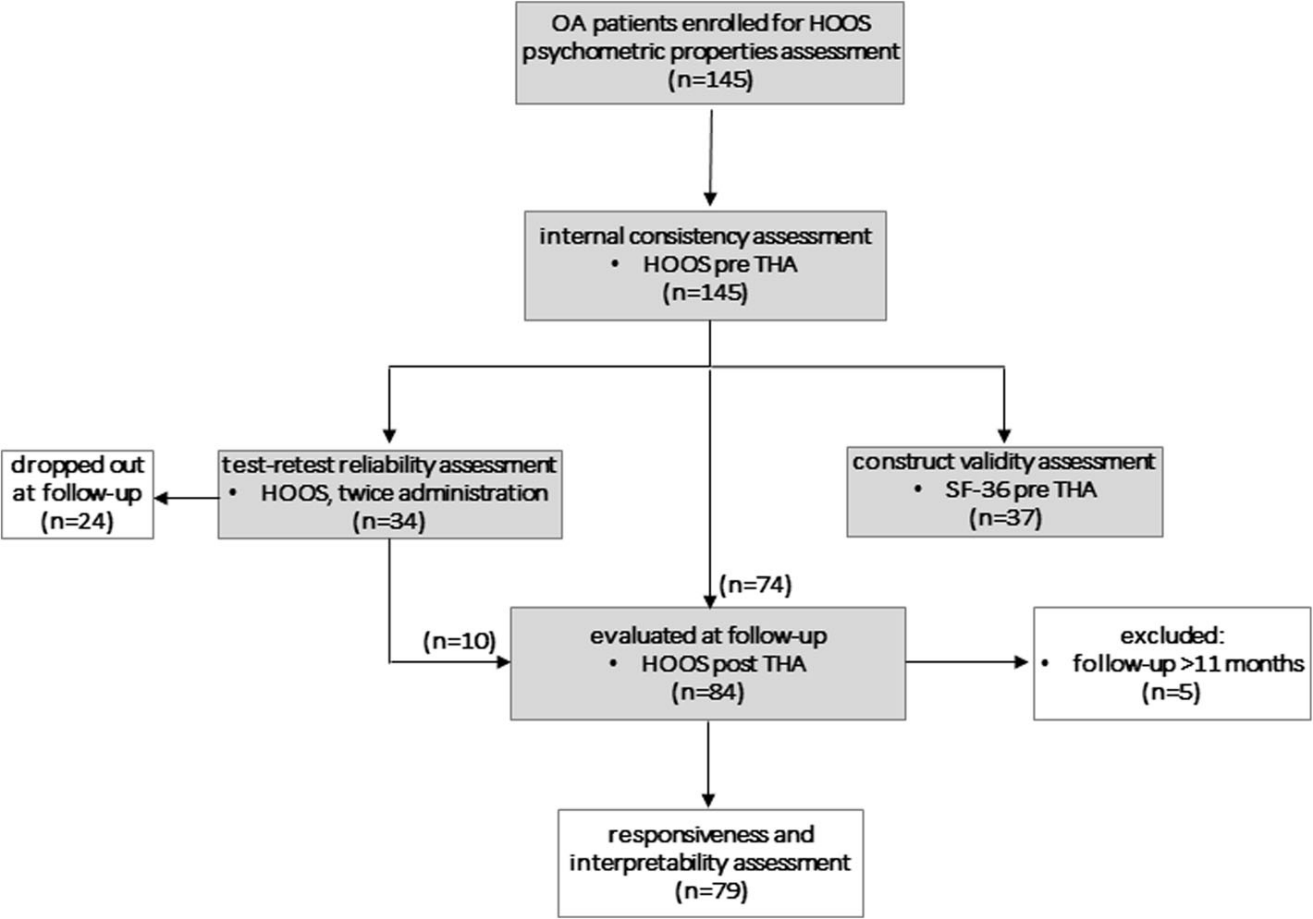
Flow-chart of the validation process, and involved patients for each stage

A total of 145 patients (mean age = 65.7 ± 11.6, range 34–89, 58.6% women) admitted in the orthopaedic units of five Italian hospitals collaborating with RIAP were enrolled in an observational prospective multicentre study. The inclusion criteria were: age ≥ 18 years, diagnosis of hip OA, and indication for primary THA. Patients not legally competent or not able, due to their health status, or having a diagnosis of fracture, or indication for revision surgery were excluded. The enrolled patients were duly informed and their written consent to participate was collected. They were asked to fill out the Italian HOOS before surgery, to assess internal consistency. Among them, 34 agreed to fill out the HOOS one more time to evaluate test-retest reliability (5–21 days from the first administration). To assess convergent validity, a subset of 37 patients was requested to fill out the SF-36. Out of 108 patients eligible for follow-up, 24 dropped out (22.22%). Finally, 84 patients were assessed at follow-up (5–25 months). To evaluate HOOS responsiveness and interpretability, only the ones that had filled out the questionnaires within 11 months after surgery (79) were included (Fig. 1). The preoperative HOOS compilation was performed during pre-hospitalization (82.1%) or at admission (17.9%), while postoperative compilation was performed in the outpatient department setting during follow-up assessment.

#### Questionnaires

##### HOOS

HOOS was firstly developed in Sweden in 2003 from the Knee injury and Osteoarthritis Outcome Score (KOOS). It is a self-administered questionnaire and consists of 40 items divided into five subscales assessing five distinct patient-relevant dimensions: Pain (P), Symptoms (S), Activity of Daily Living (ADL), Sport and Recreation Function (Sport/Rec) and Hip related Quality of Life (QoL). The patients can express their opinion through standardized response options based on the five-point Likert scale (none, mild, moderate, severe, extreme); each answer is scored ranging from zero (no problems) to four (extreme problems). A normalized score (100 indicating no symptoms and zero indicating extreme symptoms) is calculated for each subscale (100-subscale average/4*100) and can be plotted as an outcome profile [3].

##### SF-36

SF-36 [19] is a widespread, validated psychometric generic questionnaire on HRQoL, also available in Italian [20]. It groups 36 items into eight multi-item scales: Physical Functioning (PF), Role-Physical (RP), Bodily Pain (BP), General Health (GH), Vitality (VT), Social Functioning (SF), Role-Emotional (RE) and Mental Health (MH). For each subscale, a score can range from zero (worst possible health status) to 100 (best possible health status), following the standard SF-36 scoring algorithms. The SF-36 subscales scores were computed using the algorithm for the Statistical Package for the Social Sciences (SPSS), available from the Mario Negri Institute for Pharmacological Research website [21].

### Assessment of psychometric properties

#### Internal consistency

Internal consistency is defined as “the degree of the interrelatedness among the items” [13]. It was evaluated using Cronbach’s alpha coefficient. A coefficient ≥ 0.70 was considered satisfactory [4].

#### Test-retest reliability

Test-retest reliability is defined as “the extent to which scores for patients who have not changed are the same for repeated measurement […] over time” [13]. It was assessed using Pearson’s correlation coefficient and intra-class correlation coefficient (ICC, two-way random effect model, assuming a single measurement and absolute agreement with 95% confidence interval). ICC values ≥0.80 expressed good reliability [4, 5].

#### Convergent validity

Convergent validity is part of the construct validity. Construct validity is defined as “the degree to which the scores of an HR-PRO instrument are consistent with hypotheses based on the assumption that the HR-PRO instrument validly measures the construct to be measured” [13] and is the combination of convergent validity between conceptually similar items/domains of the questionnaires compared, and divergent (or discriminant) validity between dissimilar items or domains [22]. The convergent validity was tested by using Spearman’s rank correlation coefficient between the results of each HOOS and SF-36 subscales. Spearman’s correlation coefficients (rho) > 0.50, 0.35–0.50, and < 0.35 were considered strong, moderate and weak correlations, respectively [6, 7, 9]. As reported by Scalone et al. [23], to be considered relevant for convergence, the correlation coefficients between equivalent domains are required to be higher than 0.2 and statistically significant. The a priori hypothesis was that strong correlations would be observed between the subscales measuring similar domains (i.e. SF-36 BP vs HOOS Pain, SF-36 PF vs HOOS ADL).

#### Responsiveness

Responsiveness is defined as “the ability of an HR-PRO instrument to detect change over time in the construct to be measured” [13]. This property measures the capability of the questionnaire to capture real changes, i.e. it measures the sensitivity to change if real changes occur. It was tested by comparing the pre- and post-THA scores of every HOOS subscale using the Wilcoxon signed rank test for paired data, considering a level of significance < 0.01 [6, 7]. Moreover, the Standardized Response Mean (SRM), i.e. the mean change between baseline and follow-up divided by the SD of this change, and the Effect Size (ES), i.e. the mean score change between baseline and follow-up divided by the SD of the baseline values were calculated [4], considering them large when ≥0.80 [2, 24].

### Assessment of interpretability and acceptability

#### Interpretability

According to the COSMIN study [13], interpretability is not considered a measurement property, but an important characteristic of a measurement instrument. It is defined as “the degree to which one can assign qualitative meaning - that is, clinical or commonly understood connotations – to an instrument’s quantitative scores or change in scores”. It was assessed by calculating floor and ceiling effects and kurtosis and asymmetry indices for each HOOS subscale pre- and post-THA. A priori values for floor and ceiling effects (> 15% of participants responding with the lowest/highest possible score, respectively) [4–6, 25] and kurtosis and asymmetry indices (≤ |1.0|) were defined [26, 27].

#### Acceptability

Acceptability of the Italian HOOS was investigated in pre-THA by measuring the average time needed to fill out the HOOS, the proportion of missing answers, and the self-confidence of patients in compilation. A compilation time < 15 min was considered satisfactory [3, 10]. Referring to the proportion of missing answers and patients needing support in compilation, a < 5% value was assumed to be acceptable [10].

#### Missing data

Missing data were treated as follows. HOOS: the HOOS Scoring instructions were applied [28]; SF-36: the algorithm developed by the Mario Negri Institute for Pharmacological Research [21] according to the SF-36 instructions was applied.

#### Statistics

SPSS version 23.0 was used for statistical analyses. *P* values of < 0.05 were considered significant.

## Results

### Development of the Italian version of the HOOS: translation and cross-cultural adaptation

Some difficulties arose only when translating items P4 “Walking on a flat surface” and A12 “Lying in bed (turning over, maintaining hip position)”. No patients reported having met problems in reading and understanding the HOOS items.

### Assessment of psychometric properties

#### Internal consistency

Table 1 shows the internal consistency for the patients enrolled in the study (145). Cronbach’s alpha ranged from 0.70 to 0.96.

**Table 1:** Internal consistency of the five HOOS subscales, *n* = 145

HOOS subscales	Cronbach’s alpha coefficient (95% CI)
Symptoms	0.70 (0.62–0.77)
Pain	0.89 (0.86–0.92)
ADL	0.96 (0.95–0.97)
Sport/Rec	0.89 (0.85–0.91)
QoL	0.80 (0.74–0.85)

*ADL* Activity of Daily Living*, Sport/Rec* Sports and Recreational activities*, QoL* Quality of Life

#### Test-retest reliability

The measurement of test-retest reliability performed on a subset of 34 patients is showed in Table 2. For all the Italian HOOS subscales, Pearson’s correlation coefficient ranged from 0.83–0.91 and ICC from 0.81 to 0.91.

**Table 2:** Test-retest reliability for the five HOOS subscales, *n* = 34

HOOS subscales	Pearson’s coefficient	ICC (95% CI)
Symptoms	0.91	0.91 (0.83–0.96)
Pain	0.91	0.91 (0.82–0.95)
ADL	0.89	0.87 (0.71–0.94)
Sport/Rec	0.84	0.81 (0.58–0.91)
QoL	0.83	0.81 (0.62–0.90)

*ADL* Activity of Daily Living*, Sport/Rec* Sports and Recreational activities*, QoL* Quality of Life

#### Convergent validity

Thirty-seven patients were asked to fill out the SF-36 to evaluate convergent validity. Spearman’s correlation coefficient values > 0.50 were found for subscales referred to similar measures (i.e. BP vs each HOOS subscale rho> 0.60; SF vs ADL, rho = 0.55; PF and MH vs Sport/Rec, rho = 0.57). Lower values (rho ≤0.22) were found between subscales referred to different aspects of the construct (i.e. RE vs each HOOS subscale) (Table 3).

**Table 3:** Convergent validity: Spearman’s correlation coefficients between each subscale of the Italian HOOS and SF-36, *n* = 37

	*HOOS subscales*				
	Symptoms	Pain	ADL	Sport/Rec	QoL
SF-36 subscales					
PF	**0.42**	**0.58**	**0.65**	**0.57**	**0.46**
RP	0.28	**0.37**	**0.46**	**0.55**	0.32
BP	**0.61**	**0.75**	**0.73**	**0.66**	**0.69**
GH	0.21	**0.34**	**0.38**	0.29	0.18
VT	**0.36**	**0.48**	**0.47**	**0.47**	**0.42**
SF	**0.49**	**0.61**	**0.55**	**0.55**	**0.45**
RE	0.23	0.15	0.17	0.24	0.13
MH	**0.45**	**0.48**	**0.52**	**0.57**	**0.42**

Bold figures indicate significant correlation (*p* < 0.05)

*PF* Physical Functioning, *RP* Role-Physical, *BP* Bodily Pain, *GH* General Health, *VT* Vitality, *SF* Social Functioning, *RE* Role-Emotional, *MH* Mental Health, *ADL* Activity of Daily Living*, Sport/Rec* Sports and Recreational activities*, QoL* Quality of Life

#### Responsiveness

**Fig. 2:**
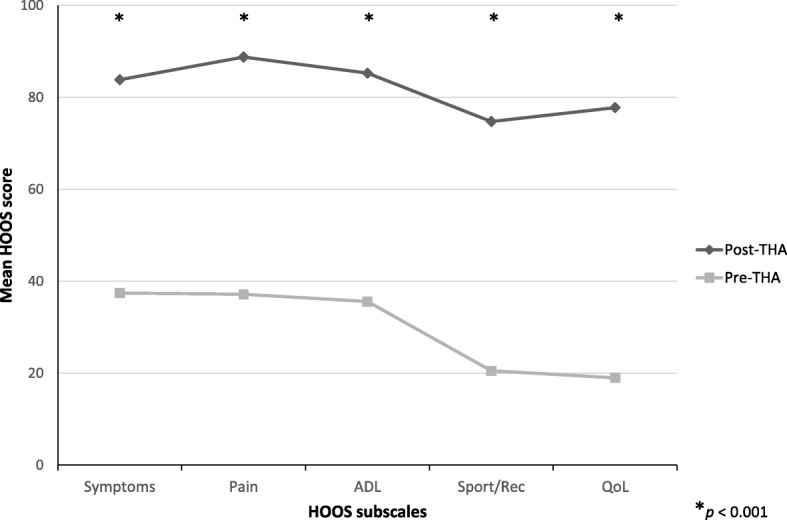
HOOS profiles before (Pre) and after (Post) THA (*n* = 79). The Wilcoxon signed rank test was used to compare the Pre and Post data of each subscale of the Italian HOOS. This scale is 0–100, worst to best. *: *p* < 0.001

Responsiveness was tested by comparison of the preoperative and postoperative scores of 79 patients. All HOOS subscales scores improved significantly after THA (*p* < 0.001) as determined using Wilcoxon signed rank test (Fig. 2). ES ranged from 2.72 to 3.71 and SRM from 1.84 to 2.38 (Table 4).

**Table 4:** Responsiveness of the Italian HOOS subscales. Mean and standard deviation (SD) before (Pre) and after (Post) THA; Wilcoxon signed rank test statistical significance (*p*-value); Effect Size (ES); Standardized Response Mean (SRM), *n* = 79

HOOS subscales	Mean	SD	*p*-value	ES	SRM
Symptoms					
Pre	37.44	15.56	< 0.001	2.93	2.09
Post	83.84	15.83			
Pain					
Pre	37.17	15.54	< 0.001	3.32	2.38
Post	88.78	14.48			
ADL					
Pre	35.58	17.16	< 0.001	2.90	2.19
Post	85.27	15.78			
Sport/Rec					
Pre	20.51	19.94	< 0.001	2.72	1.84
Post	74.76	23.23			
QoL					
Pre	18.99	15.84	< 0.001	3.71	2.03
Post	77.77	25.41			

*ADL* Activity of Daily Living, *Sport/Rec* Sports and Recreational activities, *QoL* Quality of Life

### Assessment of interpretability and acceptability

#### Interpretability

Data from 79 patients were assessed to measure interpretability. Floor effect was found in preoperative assessment in subscales Sport/Rec (26.92%) and QoL (22.78%). Ceiling effect was found in postoperative assessment in all the subscales, particularly in QoL (40.51%). The values of skewness and kurtosis were lower or slightly above unity, except for kurtosis of the Pain subscale in postoperative assessment (2.85) (Table 5).

**Table 5:** Interpretability of Italian HOOS. Skewness and kurtosis values, and floor and ceiling effects, before (Pre) and after (Post) THA, *n* = 79

	Skewness		Kurtosis		Floor effect (%)		Ceiling effect (%)	
HOOS subscale	Pre	Post	Pre	Post	Pre	Post	Pre	Post
Symptoms	0.44	−0.81	0.75	−0.22	0.00	0.00	0.00	27.85
Pain	0.70	−1.75	0.86	2.85	0.00	0.00	0.00	29.11
ADL	1.03	−1.17	1.82	0.56	0.00	0.00	0.00	15.19
Sport/Rec	1.18	−0.69	1.65	−0.21	26.92	0.00	0.00	28.21
QoL	0.74	−0.83	0.34	−0.65	22.78	0.00	0.00	40.51

*ADL* Activity of Daily Living, *Sport/Rec* Sports and Recreational activities, *QoL* Quality of Life

#### Acceptability

Acceptability was tested on the preoperative administration of the HOOS (145 patients). On average, the time to fill out the questionnaire was 12 min. The proportion of missing data was 1.16% (Symptoms 0.28%, Pain 1.17%, ADL 1.50%, and Sport/Rec 1.90%). No missing data were registered for QoL. The item A13 “Getting in/out of bath” (derived from WOMAC) recorded the highest number of missing answers (4.94%). Some elderly patients (3%) requested to be assisted by a relative or, more rarely, an operator to fill out the questionnaire.

## Discussion

In the present study, the Italian version of HOOS was developed and the psychometric properties were assessed. Translation into Italian and cross-cultural adaptation of the English version posed some difficulties that were resolved anyway. In the end, no patient reported issues in reading and understanding the HOOS items. The psychometric properties showed that the Italian version of HOOS is a valid and reliable tool to assess outcomes in OA patients undergoing THA.

The results of the assessment of the internal consistency were good for all subscales and were comparable to those observed in other language versions of the HOOS [4–7, 9] suggesting good homogeneity. Cronbach’s alpha ADL subscale scored the highest value (0.96), according to the French (0.94) [4], Dutch (THA) (0.95) [5], Korean (0.96) [6] and original HOOS (0.96) [1] validation studies. As reported by other authors [5, 6], values of Cronbach’s alpha greater than 0.90 mean that some of the 17 items of the ADL subscale could be removed because they may be redundant. The Symptoms subscale presented the lowest value (0.70) of the Cronbach’s alpha. Although acceptable and higher than that found for the French version (0.66) [4], it improved to 0.77 when item S1 (Do you feel grinding, hear clicking or any other type of noise from your hip?) was excluded from the analysis. This result is closer to the value measured in another study (0.75) [6]. In fact, this item presented a higher response variability compared to the others of the same subscale. In addition, its lower consistency with the other items of the Symptom’s subscale might be due to the fact that two different types of perception are simultaneously investigated (a hip joint internal noise compared to a physical difficulty).

Similarly to the results found in French (0.83–0.89) [4], Dutch (THA) (0.75–0.89) [5] and original HOOS (0.78–0.91) [1] validation studies, test-retest reliability ICC ranged from 0.81 to 0.91, showing that the Italian HOOS appears to be stable over time.

The a priori hypothesis for convergent validity was confirmed. Likewise it was observed in other language versions [2, 5, 6], all the HOOS subscales showed the highest correlations with the SF-36 subscale BP, confirming the important role of this component in defining perceived health status in OA patients undergoing THA. The correlation between PF and ADL measured for the Italian HOOS (0.65) is strong, as already observed in the Japanese (0.61) [7], Dutch (0.72) [5], Korean (0.80) [6] and in the original HOOS (0.66) [2] validation studies. The HOOS subscales showed mostly moderate and weak correlations with SF-36 VT and MH and RP, GH, RE, respectively.

Responsiveness to clinical change is an important property of outcome measures. In the present study, all the subscales showed good responsiveness and all subscales scores improved postoperatively, compared to the preoperative ones (*p* < 0.001). These results are consistent with what was clinically observed for THA since its introduction in the 1960s. In his study [29], Harris defined THA the most successful surgery for OA patients, and Learmonth described it as “the operation of the century” [30]. Initially restricted to either elderly and infirm people or individuals with limitations affecting locomotion, today THA is mainly intended for individuals who deem unacceptable a compromise in quality of life [30]. The ES (2.72–3.71) and SRM (1.84–2.38) of the Italian HOOS were comparable to the values measured in the French (ES: 1.97–3.24; SRM: 1.54–2.08) [4] and Chinese (ES: 2.53–3.33; SRM: 2.16–3.12) [10] validation studies. High values of ES and SRM were showed by all the language validation studies of HOOS that enrolled patients undergoing THA [2, 4, 7, 10], a procedure that usually determines a great improvement in clinical outcomes.

Floor effect was observed in preoperative administration for subscales Sport/Rec (26.92%) and QoL (22.78%). Ceiling effect was found in post-THA assessment for the subscales Symptoms (27.85%), Pain (29.11%), Sport/Rec (28.21%), and QoL (40.51%). According to the French study [4], the only observing a floor effect (17.8%), the markedly lower scores measured in the two subscales not included in the WOMAC (Sport/Rec and QoL) might be related to the age of patients (French: 67.5 years [50–81], Italian: 65.7 [34–89]). Ceiling effect was observed in other studies for Pain (HOOS validation: 19% [2], Japanese: 44% [7]) and Symptoms (Japanese: 29% [7]) but not for Sport/Rec and QoL subscales. This effect was explored further by performing a subgroup analysis based on age, gender and hospital. While no significant differences were observed for age and gender, the analysis by hospitals highlighted for a single hospital postoperative values in all the subscales significantly higher than those measured in the other hospitals. After excluding this hospital from the analysis, Pain remained the only subscale showing a ceiling effect (15.38%). However, it has to be taken into account that, considering the increased effectiveness of THA, high ceiling effect can be expected and that the criterion of having the best possible score in less than 15% of respondents might be too restrictive [31–33]. Asymmetry and kurtosis values were generally less than unity showing a good enough discriminant capacity of the subscales.

The Italian HOOS proved to be acceptable, easily understood and could be self-administered in about 12 min. The item that registered the highest number of missing answers (4.94%) was A13 “Getting in/out of bath” (derived from WOMAC), in most of the cases explained by the patients because they didn’t have a bathtub in their home. At the time WOMAC was developed, most of the houses had bathtubs. Today it is different as many houses are often equipped only with showers. As Ewa Roos already did while adapting WOMAC to the Swedish setting [34], and as it is in the Danish, Dutch, Norwegian, Polish and Swedish HOOS translations [35], item A13 will be corrected also in the final Italian HOOS, substituting it with “Getting in/out of bath/shower”, after the validation study will be completed (see Additional file 1).

Five orthopaedic units of hospitals from different geographical areas were recruited in this multicentre study to collect data from OA patients undergoing THA in Italy. Data collection raised some difficulties, therefore patients were arranged in several subgroups and different outcomes were assessed. Even if collecting data from several hospitals might be considered a limitation for the study, it was a valuable opportunity to test the Italian HOOS in a real rather than experimental setting. For example, while assessing responsiveness and interpretability, it was possible to detect that in one single hospital unit a response bias had been introduced leading to higher values for postoperative measurements. When that unit was excluded from the data analysis, both ES and ceiling effects shifted to values closer to those of other studies. This led to the awareness that to optimize a HR-PRO instrument’s properties, the burden to respondents and healthcare personnel in completing and administering questionnaires should be taken into account [11]. In addition, the questionnaire administration should be standardized to avoid possible response bias. A further limitation is that only a generic HRQoL questionnaire was used to test convergent validity. Although the results obtained are consistent with what expected, the use of a disease-specific questionnaire on hip dysfunction besides the SF-36 might have assessed this psychometric property in a more specific way.

To the best of our knowledge, this is the first study adapting and validating the HOOS in Italy. Translating it posed a few difficulties regarding the wording related to items P4 (Walking on a flat surface) and A12 (Lying in bed (turning over, maintaining hip position)). As to P4, it was discussed if “flat” meant “having a continuous horizontal surface” or “having a relatively smooth or even surface”. The latter would have been a counterpart for item P10 “Walking on an uneven surface” where “uneven” is used. In fact, the back translator into English used the word “even” for P4. In the end, the Italian word “piana” was selected, which is similarly ambiguous and was also used in the Italian WOMAC [18]. As to A12, the comma was discarded and the sentence interpreted in a way that lead to this back translation “turning over while keeping your hip still”. The question was if in the original version these two actions were assumed to be disjoined (“turning over in bed” or “maintaining hip position still while lying in bed”). All these cross-language and transcultural issues were discussed and it was decided that the first part of the item (“lying in bed”) was already indicating a still position, therefore the sentence without the comma was kept.

Issues concerning items P4, A12 and A13 were shared with one of the original HOOS authors, Ewa Roos, and other researchers that are currently working on a project aiming to develop a short version of the HOOS. On 17 February 2017, they emailed the principal investigator of this study (MT) asking to be provided with any feedback about the ease or difficulty of translating the items into Italian. Since the HOOS is an international instrument, they considered ideal to incorporate as much international perspective as possible about the items into the selection process. In their opinion, in fact, it would be a huge mistake to include in a HOOS short form an item that is difficult to translate into other languages (Barbara Gandek and Ewa Roos, personal communication 2017).

## Conclusions

The English HOOS was translated into Italian and transculturally adapted, in accordance with international guidelines. The Italian HOOS showed to be a reliable tool to assess patient-related outcomes and effectiveness of clinical interventions in OA patients undergoing THA.

Its ability to detect changes over time and its easiness suggest it could be a useful tool to be implemented within the RIAP routine data collection.

## Additional file

Additional file 1: Hip disability and Osteoarthritis Outcome Score (HOOS), Italian version, LK 2.0, March 2017. (PDF 303 kb)

## Abbreviations

ADL: Activity of Daily Living
BP: Bodily Pain
ES: Effect Size
GH: General Health
HOOS: Hip disability and Osteoarthritis Outcome Score
HR-PRO: Health-Related Patient-Reported Outcomes
HRQoL: Health-related quality of life
ICC: Intra-class correlation coefficient
ISS: Istituto Superiore di Sanità
KOOS: Knee injury and Osteoarthritis Outcome Score
MH: Mental Health
OA: Osteoarthritis
P: Pain
PF: Physical Functioning
PROMs: Patient-reported outcomes measures
QoL: Quality of Life
RE: Role-Emotional
RIAP: Italian Arthroplasty Registry project
RP: Role-Physical
S: Symptoms
SD: Standard Deviation
SF: Social Functioning
SF-36: 36-Item Short Form Health Survey
Sport/Rec: Sport and Recreation Function
SPSS: Statistical Package for the Social Sciences
SRM: Standardized Response Mean
THA: Total Hip Arthroplasty
VT: Vitality
WOMAC: Western Ontario and McMaster Universities Osteoarthritis Index
