# Supplementary material for: Cross-cultural adaptation and validation of the Italian version of the Hip disability and Osteoarthritis Outcome Score (HOOS)
Source: Health Qual Life Outcomes. 2018 Jun 4;16:115. doi: 10.1186/s12955-018-0935-6 (PMC5987663; doi:10.1186/s12955-018-0935-6)
Supplement: Supplementary file 1 — Hip disability and Osteoarthritis Outcome Score (HOOS), Italian version, LK 2.0, March 2017. (PDF 303 kb) [file 12955_2018_935_MOESM1_ESM.pdf]

QUESTIONARIO HOOS PER PAZIENTI CON PATOLOGIE DELL'ANCA

Data: ______/______/______ Data di nascita: ______/______/______

Nome: ____

ISTRUZIONI
Questo questionario è stato predisposto per conoscere il Suo parere sullo stato della Sua anca. Queste informazioni possono aiutarci a comprendere cosa Lei pensa del Suo disturbo e quanto Lei sia in grado di compiere le comuni attività della vita quotidiana.
Risponda a ciascuna domanda, mettendo una crocetta nella casella corrispondente (solo una risposta per ciascuna domanda). Se nessuna delle risposte dovesse sembrarLe appropriata, scelga quella che più si avvicina alla Sua situazione.

Sintomi

Le domande che seguono riguardano i sintomi e le difficoltà che ha provato all'anca **nel corso dell'ultima settimana**.

S1. Scrosci, scatti o altri tipi di rumore

Mai	Raramente	Qualche volta	Spesso	Sempre
❑	❑	❑	❑	❑

S2. Difficoltà nel divaricare al massimo le gambe

Nessuna	Lieve	Moderata	Grave	Estrema
❑	❑	❑	❑	❑

S3. Difficoltà a fare passi lunghi mentre cammina

Nessuna	Lieve	Moderata	Grave	Estrema
❑	❑	❑	❑	❑

Rigidità

Le domande che seguono riguardano il grado di rigidità che ha provato all'anca **nel corso dell'ultima settimana**. La rigidità è una sensazione di limitazione o minore facilità nel muovere l'anca.

S4. Quanto intensa è la rigidità della sua anca subito dopo il risveglio al mattino?

Nessuna	Lieve	Moderata	Grave	Estrema
❑	❑	❑	❑	❑

S5. Quanto intensa è la rigidità della sua anca dopo essere stato seduto, disteso o aver riposato nel corso della giornata?

Nessuna	Lieve	Moderata	Grave	Estrema
❑	❑	❑	❑	❑

Dolore

P1. Quanto frequentemente le fa male l’anca?

Mai	Mensilmente	Settimanalmente	Quotidianamente	Sempre
❑	❑	❑	❑	❑

Quale è stata l’intensità del dolore che ha provato all'anca **nell'ultima settimana** durante le seguenti attività?

P2. Estendere completamente l’anca

Nessuna	Lieve	Moderata	Grave	Estrema
❑	❑	❑	❑	❑

P3. Flettere completamente l’anca

Nessuna	Lieve	Moderata	Grave	Estrema
❑	❑	❑	❑	❑

P4. Camminare su una superficie piana

Nessuna	Lieve	Moderata	Grave	Estrema
❑	❑	❑	❑	❑

P5. Salire o scendere le scale

Nessuna	Lieve	Moderata	Grave	Estrema
❑	❑	❑	❑	❑

P6. Di notte, a letto

Nessuna	Lieve	Moderata	Grave	Estrema
❑	❑	❑	❑	❑

P7. Stare seduto o sdraiato

Nessuna	Lieve	Moderata	Grave	Estrema
❑	❑	❑	❑	❑

P8. Stare in piedi in posizione eretta

Nessuna	Lieve	Moderata	Grave	Estrema
❑	❑	❑	❑	❑

P9. Camminare su una superficie rigida (asfalto, cemento, ecc.)

Nessuna	Lieve	Moderata	Grave	Estrema
❑	❑	❑	❑	❑

P10. Camminare su una superficie irregolare

Nessuna	Lieve	Moderata	Grave	Estrema
❑	❑	❑	❑	❑

Funzione, attività della vita quotidiana

Le domande che seguono riguardano le sue capacità funzionali, in particolare la sua abilità nel muoversi e prendersi cura di sé. Per ciascuna delle seguenti attività indichi per favore il grado di difficoltà provato **nell'ultima settimana** a causa dei problemi all'anca.

A1. Scendere le scale

Nessuna	Lieve	Moderata	Grave	Estrema
❑	❑	❑	❑	❑

A2. Salire le scale

Nessuna	Lieve	Moderata	Grave	Estrema
❑	❑	❑	❑	❑

A3. Alzarsi da seduto

Nessuna	Lieve	Moderata	Grave	Estrema
❑	❑	❑	❑	❑

A4. Stare in piedi

Nessuna	Lieve	Moderata	Grave	Estrema
❑	❑	❑	❑	❑

A5. Piegarsi verso il pavimento/raccogliere un oggetto

Nessuna	Lieve	Moderata	Grave	Estrema
❑	❑	❑	❑	❑

A6. Camminare su una superficie piana

Nessuna	Lieve	Moderata	Grave	Estrema
❑	❑	❑	❑	❑

A7. Salire e/o scendere da un'automobile

Nessuna	Lieve	Moderata	Grave	Estrema
❑	❑	❑	❑	❑

A8. Andare a fare spese

Nessuna	Lieve	Moderata	Grave	Estrema
❑	❑	❑	❑	❑

A9. Mettersi i calzini o le calze

Nessuna	Lieve	Moderata	Grave	Estrema
❑	❑	❑	❑	❑

A10. Alzarsi dal letto

Nessuna	Lieve	Moderata	Grave	Estrema
❑	❑	❑	❑	❑

A11. Togliersi i calzini o le calze

Nessuna	Lieve	Moderata	Grave	Estrema
❑	❑	❑	❑	❑

A12. Stare sdraiato a letto (girarsi tenendo ferma l'anca)

Nessuna	Lieve	Moderata	Grave	Estrema
❑	❑	❑	❑	❑

A13. Entrare e/o uscire dalla vasca da bagno/doccia

Nessuna	Lieve	Moderata	Grave	Estrema
❑	❑	❑	❑	❑

A14. Stare seduto

Nessuna	Lieve	Moderata	Grave	Estrema
❑	❑	❑	❑	❑

A15. Sedersi e/o alzarsi dal water

Nessuna	Lieve	Moderata	Grave	Estrema
❑	❑	❑	❑	❑

A16. Fare lavori domestici pesanti (spostare scatole pesanti, lavare pavimenti, ecc.)

Nessuna	Lieve	Moderata	Grave	Estrema
❑	❑	❑	❑	❑

A17. Fare lavori domestici leggeri (cucinare, spolverare, ecc.)

Nessuna	Lieve	Moderata	Grave	Estrema
❑	❑	❑	❑	❑

Funzione fisica, attività sportive e del tempo libero

Le domande che seguono riguardano le sue capacità funzionali quando svolge attività che richiedono un impegno maggiore. Faccia riferimento al grado di difficoltà provato a causa dell'anca **nell'ultima settimana**.

SP1. Accovacciarsi

Nessuna	Lieve	Moderata	Grave	Estrema
❑	❑	❑	❑	❑

SP2. Correre

Nessuna	Lieve	Moderata	Grave	Estrema
❑	❑	❑	❑	❑

SP3. Girarsi facendo perno sulla gamba

Nessuna	Lieve	Moderata	Grave	Estrema
❑	❑	❑	❑	❑

SP4. Camminare su una superficie irregolare

Nessuna	Lieve	Moderata	Grave	Estrema
❑	❑	❑	❑	❑

Qualità della vita

Q1. Con quale frequenza si rende conto del suo problema all’anca?

Mai	Mensilmente	Settimanalmente	Quotidianamente	Sempre
❑	❑	❑	❑	❑

Q2. Ha modificato il Suo stile di vita per evitare attività potenzialmente dannose per la Sua anca?

Per niente	Poco	In parte	Molto	Moltissimo
❑	❑	❑	❑	❑

Q3. Quanto è preoccupato dalla mancanza di sicurezza della sua anca?

Per niente	Poco	In parte	Molto	Moltissimo
❑	❑	❑	❑	❑

Q4. In generale, quanto è in difficoltà a causa della sua anca?

Per niente	Poco	In parte	Molto	Moltissimo
❑	❑	❑	❑	❑

La ringraziamo molto per aver risposto a tutte le domande del questionario!
